# 

**Published:** 1895-11

**Authors:** 


### CONTENTS.

ORIGINAL ARTICLES—

The Necessity of Complete Extirpation of Tumors and the Importance of Rapid Cicatrization of the Wound. By Frederick Holme Wiggin, M. D., New York .......... 551

Clinical Study in Typhoid Fever. By D. E. Parsons, M. D., Oakland, Me .......... 558

Tendon Grafting: A New Operation for Deformities Following Infantile Paralysis, With Report of a Successful Case. By Samuel Milliken, M. D., New York .......... 565

New York Letter .......... 566

SOCIETY REPORTS—

Tri-State Medical Society of Georgia, Alabama and Tennessee... 570

SELECTIONS AND ABSTRACTS—

Ammonol in Malarial Paroxysm. By Cyrus Edson, M. D., New York .......... 578

Rheumatism and its Successful Treatment. By William C. Wile, A. M., M. D., LL. D., Danbury, Conn ........ 580
Treatment of Chancere ........ 582

EDITORIAL—

Philadelphia Academy of Surgery.—The Samuel D. Gross Prize. 584
Tri-State Medical Association of Georgia, Alabama and Tennessee ........ 584
Notes ........ 587
Book Reviews ........ 588
Special Notes ........ 592
Prescriptions ........ 590

